# Alterations of the iNKT cell compartment in brain-injured patients

**DOI:** 10.1186/s13054-019-2518-2

**Published:** 2019-06-28

**Authors:** Allan Patinec, Jézabel Rocher, Mickael Vourc’h, Antoine Roquilly, Karim Asehnoune, Jacques Le Pendu

**Affiliations:** 1 CRCINA, Inserm, Université d’Angers, Université de Nantes, 44007 Nantes, France; 2 Laboratoire EA3826 Thérapeutiques cliniques et expérimentales des infections IRS2 Nantes Biotech, Université de Nantes, Nantes, France; 3 CHU Nantes, Pôle anesthésie réanimations Service d’anesthésie réanimation chirurgicale, Hôtel Dieu, F-44093 Nantes, France

**Keywords:** Brain injury, Nosocomial pneumonia, iNKT cells, CD1d, Immunodepression, Beta2 adrenergic receptor

## Abstract

**Background:**

Brain injury (BI) induces a state of immunodepression leading to pneumonia. We investigated the invariant natural killer T (iNKT) cell compartment.

**Methods:**

This is an observational study in two surgical intensive care units (ICUs) of a single institution and a research laboratory. Clinical data and samples from a prospective cohort were extracted. Severe brain-injured patients (*n* = 33) and sex- and age-matched healthy donors (*n* = 40) were studied.

**Results:**

We observed the presence of IL-10 in serum, a loss of IFN-γ and IL-13 production by peripheral blood mononuclear cells (PBMCs) following IL-2 stimulation, and downregulation of HLA-DR expression on both monocytes and B cells early after BI. Inversely, CD1d, the HLA class I-like molecule involved in antigen presentation to iNKT cells, was over-expressed on patients’ monocytes and B cells. The antigen-presenting activity to iNKT cells of PBMCs was increased in the patients who developed pneumonia, but not in those who remained free of infection. Frequencies of iNKT cells among PBMCs were dramatically decreased in patients regardless of their infection status. Following amplification, an increased frequency of CD4+ iNKT cells producing IL-4 was noticed in the group of patients free of infection compared with those who became infected and with healthy donors. Finally, serum from BI patients inhibited the iNKT cells’ specific response as well as the non-specific IL-2 stimulation of PBMCs, and the expression of the beta-2 adrenergic receptor was elevated at the surface of patients T lymphocytes.

**Conclusions:**

We observed severe alterations of the iNKT cell compartment, including the presence of inhibitory serum factors. We demonstrate for the first time that the decreased capacity to present antigens is not a generalized phenomenon because whereas the expression of HLA-DR molecules is decreased, the capacity for presenting glycolipids through CD1d expression is higher in patients.

**Electronic supplementary material:**

The online version of this article (10.1186/s13054-019-2518-2) contains supplementary material, which is available to authorized users.

## Introduction

Susceptibility to nosocomial infections, mainly pneumonia [1–3] after an acute brain injury (BI) [4], is correlated with an acute immunodepression [5–7]. Primary (stroke) or secondary cerebral ischemia following traumatic brain injury (TBI) or spontaneous sub-arachnoid hemorrhage (SAH) is a major factor of central nervous system injury-induced immunodepression. This systemic immunodepression may be explained through a sympathetic storm [6, 8]. Using a mouse stroke model, Wong et al. reported that invariant natural killer T cells (iNKT) and catecholamines play a major role in the immunodepression [9]. These authors observed that iNKT cells produced more anti-inflammatory cytokines (IL-10) after induction of BI, whereas their secretion of pro-inflammatory cytokines decreased (IFN-γ, IL-12). An efficient anti-infectious immunity could be restored when iNKT cells were specifically stimulated with their canonical ligand α-galactosylceramide (α-GalCer). Moreover, either blocking the catecholamine pathway with a specific antagonist (propranolol) or using iNKT-deficient mice prevented the switch of cytokines secretion from a T_h_1 to a T_h_2 profile and restored clearance of infection after stroke.

iNKT cells are T lymphocytes characterized by an invariant T cell receptor [10]. T cell receptor (TCR) recognition is restricted by the monomorphic MHC class-I-like molecule CD1d that is expressed by antigen-presenting cells (APC) [11]. In contrast to conventional T cells which recognize peptides, the iNKT TCR reacts to self or foreign lipid antigens (bacterial lipids) loaded on CD1d. Several subsets of iNKT cells have been described based on their expression of the CD4 or CD8 molecules. They can be CD4^+^/CD8^−^, CD4^−^/CD8^+^, or CD4^−^/CD8^−^ [12], the CD4+ subset likely representing less mature cells [13, 14]. Activation of iNKT cells leads to a quick and massive release of both pro-inflammatory (T_h_1) and anti-inflammatory (T_h_2) cytokines, the CD4+ subset releasing both IL-4 and IFN-γ, while the CD4- subset releases IFN-γ preferentially [13, 14]. iNKT cells are highly versatile cells that can contribute to various types of immune responses, including anti-microbial and anti-cancer responses, but also inflammatory and autoimmune diseases. The type of response to which they contribute is largely dependent upon the context and the APCs with which they interact. They are particularly efficient to drive the first stages of innate responses [14].

We previously demonstrated that TBI and SAH displayed an immune dysfunction characterized by an attenuated granulomatous immune response ex vivo [7]. We hypothesized that the iNKT cell compartment could also play a role in immunosuppression that follows TBI or SAH. We determined (1) the number and functions of iNKT and APC and (2) the correlation between iNKT activity and subsequent infection.

## Materials and methods

### Patients and healthy volunteers

This work is part of a global study on immune dysfunctions in ICU. An institutional review board for human experimentation approved the protocol (Comité de Protection des Personnes de Nantes, authorization number AC-2008-433/French). As patients were unable to consent, written informed consent from next of kin was required for enrollment. Whenever possible, retrospective consent was obtained from patients.

Intubated patients with either a severe head trauma (TBI) or a spontaneous subarachnoid hemorrhage (Glasgow coma scale < 13 and abnormal initial CT scan) were enrolled from January 2013 to November 2013 in two French surgical ICUs of one university hospital. Midazolam and fentanyl/sufentanil were the only drugs used when deep sedation was needed. Thiopental was used in case of refractory intracranial hypertension. Control samples were collected from healthy blood donors at the Blood Transfusion Center (Etablissement Français du Sang, Nantes, France) after obtaining informed consent. The exclusion criteria were aplasia, recent chemotherapy or immunosuppressive treatment, or corticosteroid treatment, primary immune deficiency.

Nosocomial pneumonia was defined as pneumonia occurring 48 h or more after admission and not incubating at the time of admission. Pneumonia was diagnosed according to international recommendations [15], and all patients with pneumonia were treated with antibiotics. The 2 participating surgical ICUs routinely used the following strategy for VAP prevention: monitoring of the tracheal cuff pressure, semirecumbent positioning, and selective oropharyngeal antiseptic decontamination. The treatment was protocolized as previously described [16], and neuro-ICU management was carried-out according to international guidelines [17, 18]. Eighteen patients were diagnosed for early pneumonia (< 10 days [3]), whereas the other 15 did not declare nosocomial infection. Pneumonia diagnosis was always confirmed by culture from lower respiratory tract samples obtained by endotracheal aspirate, by bronchoalveolar lavage, or with a blind-protected specimen catheter (significant threshold, 10^6^ colony-forming units/mL, 10^4^ colony-forming units/mL, 10^3^ colony-forming units/mL, respectively).

### Sample collection

Blood samples were collected after ICU admission within 24 h following BI. PBMCs were obtained by gradient centrifugation following standard protocol, and serum was isolated by centrifugation and stored in liquid nitrogen or at − 80 °C until investigation, respectively.

### Flow cytometry

PBMCs and other cells were stained with anti-human mAbs: anti-CD3 FITC, anti-CD4 BV605, anti-CD8 BV421, anti-CD14 BV711, anti-CD19 BV605, anti-CD1d APC, anti-HLA-DR BV421, IFN-γ PE, and IL-4 PE (all from BD Biosciences, Vienna, Austria). APC-labeled human CD1d tetramers loaded with the α-GalCer analogue PBS-57 were obtained from the MHC Tetramer Core Facility (Emory University Vaccine Center, Atlanta, GA). Anti-human ADRB2 (AbD Serotec, Oxford, UK) was coupled with alexa fluor647 fluorochrome by using a protein labeling kit (Life Technologies, Paisley, UK). Viability was assessed with Zombie Nir viability dye (BioLegend, London, UK) or with fixable viability dye eFluor506 (eBiosciences, Vienna, Austria). The corresponding isotype control mAbs were used to assess staining specificity.

### Generation of iNKT cells

iNKT cells were enriched from PBMCs by positive selection of Vα24-Jα18 cells by magnetic beads separation (MACS Miltenyi, Paris, France). Purified cells were cultured in RPMI 1640 supplemented with 10% heat-inactivated human pooled serum from 40 donors, 2 mM glutamine, 50 U/ml penicillin, 50 μg/ml streptomycin (Gibco BRL), PHA 1 μg/ml (Sigma-Aldrich, Schnelldorf, Austria), and IL-2 300 U/ml (PeproTech, USA) in the presence of irradiated allogenic PBMCs for 1 week. Purified iNKT cells were then maintained in culture in the same medium without PHA and irradiated feeder up to 3 months. Purity of iNKT cells was assessed by flow cytometry after staining the cells with mAbs specific for CD3 and with CD1d PBS57-loaded tetramers.

### Cytokine secretion assays

PBMCs alone were cultured at cells density 1 × 10^6^/ml in 96-well culture plates at 37 °C. Mitogenic stimulation of PBMCs was performed with IL-2 (200 U/ml) for 48 h. IFN-γ and IL-13 secretions in cell supernatants were then quantified by ELISA (eBioscience).

For iNKT-specific activation with α-GalCer, antigen-presenting cells (APC) were needed. PBMCs were plated at 300,000 per well on 96-well culture plates in complete RPMI containing healthy volunteers’ pooled sera and were loaded overnight at 37 °C with 0.1 μM α-GalCer (Sigma-Aldrich). Cells were then washed twice in RPMI alone and 50,000 iNKT cells were added. In both conditions, APCs and iNKT cells were co-incubated at 37 °C in complete RPMI containing pooled sera from healthy volunteers or pooled sera from BI patients, depending on experiments. Cytokine secretion in supernatants was quantified by ELISA (eBioscience) after 48-h stimulation, as previously described [19].

### Amplification of iNKT cells from PBMCs

PBMCs from human volunteers (HV) or from patients were cultured at cell density 1 × 10^6^/ml in 24-well culture plates at 37 °C under mitogenic stimulation with IL-2 (300 UI/ml) and in the presence of α-GalCer (0.1 μM) for 10 days. Half of the medium was renewed every 3 days. At day 10, cytokine secretion was blocked with brefeldin A for 6 h, and then cells were collected to analyze intracellular cytokines by flow cytometry.

### Statistical analysis

All statistical analyses were performed with Prism-6 software (GraphPad Software). Continuous nonparametric variables are expressed as medians (interquartile range). For 2 group comparisons, the Mann-Whitney *U* test was used. The one-way analysis of variance (ANOVA) test was used for comparisons of multiple groups. Dunnett’s multiple comparison test was used as a post hoc test for intergroup comparisons. Categorical data are expressed as numbers and percentages and tested with the chi-square test or Fisher’s test. Significance was defined as *p <* 0.05.

## Results

### Clinical characteristics of patients

A total of 33 brain-injured patients were enrolled in the study. Their general characteristics are described in Table 1 and the comparison between TBI and SAH in Additional file 1**:** Table S1. The median initial GCS was 6 IQR [4–8] with a median duration of ICU stay of 15 days IQR [10–26]. Among TBI patients, 2 of them were multiple trauma with abdominal trauma for one and pelvic fracture for the other; 13 patients (39%) received norepinephrine to maintain cerebral perfusion pressure. Also, 2 patients moved to brain death at days 6 and 8. Early nosocomial pneumonia occurred in 55% of patients during their ICU stay and 18% died while in ICU. The main bacteria retrieved was methicillin-sensitive *Staphylococcus aureus*, followed by *Streptococcus pneumoniae* and *Escherichia coli*, as indicated in Additional file 2: Table S2.

**Table 1:** Characteristics of brain-injured patients

Demographic data and outcomes	Total (*n* = 33)	BI with pneumonia (*n* = 18), 55%	BI without pneumonia (*n* = 15), 45%	*p* value
Age (years)	46 [26–55]	49 [30–60]	43 [23–54]	0.2253
Male, *n (%)*	26 (79%)	14 (78%)	12 (80%)	0.7003
Initial GCS	6 [4–8]	5 [4–6.75]	7 [3.5–8]	0.4765
Barbiturate, *n (%)*	7 (21%)	3 (17%)	4 (27%)	0.2614
Corticotherapy, *n (%)*	1 (3%)	1 (6%)	0 (0%)	1
Acute respiratory distress syndrome, *n(%)*	6 (18%)	5 (28%)	1 (7%)	0.1861
Decompressive craniectomy, *n (%)*	4 (12%)	2 (11%)	2 (13%)	1
Duration of mechanical ventilation (days)	11 [7–19]	18 [11–24.7]	7 [5–12]	0.0009
ICU length of stay (days)	15 [10–26]	23 [14–34]	11 [7–16]	0.0012
Death in ICU, *n, (%)*	6 (18%)	1 (6%)	5 (33%)	0.0701

Data are given as the median [interquartile range] or *n* (%); *P* < 0.05 is considered as statistically significant

*ICU* intensive care unit, *BI* brain injury, *GCS* Glasgow coma scale

### Evidence of immunodepression in BI patients

As brain injury is known to induce an immunodepression associated with a high level of anti-inflammatory cytokines, we compared cytokine levels in serum from HV and BI patients. IL-10 was detected in sera from patients but not in HV (Additional file 3: Figure S1). We next compared the ability of BI patients and HV to secrete cytokines following a non-specific stimulation. After stimulation of PBMC with IL-2, IFN-γ, and IL-13 secretions were markedly depressed in BI patients, indicating a global unresponsiveness of patients’ immune cells (Additional file 4: Figure S2).

**Fig. 1:**
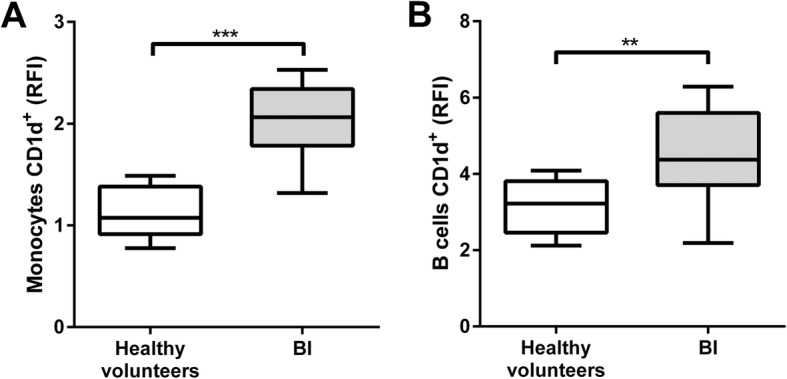
**a**, **b** Expression of CD1d on monocytes and B cells, respectively. The membrane marker, CD1d, was analyzed on stored CD14+ monocytes and CD19+ lymphocytes from 10 individual healthy volunteers (HV) and 20 individual BI patients. Data are shown as the ratio of median fluorescence intensity (RFI) obtained with the specific antibodies to those obtained with their associated control isotypes. Top and bottom whiskers represent the extreme values; boxed area represents the 25th percentile, the median, and the 75th percentile (**p < 0.01, ***p < 0.001; statistical analysis, Mann-Whitney test)

As previously described [20, 21], a major decrease of HLA-DR expression on monocytes and B cells from BI patients was observed (Additional file 5: Figure S3 and Additional file 6: Figure S4). Interestingly, CD1d expression (the molecule that presents glycolipids antigens to iNKT cell) was strongly overexpressed on both monocytes and B cells in BI patients (Fig. 1a and b). These results demonstrate that the decreased capacity to present antigens is not a generalized phenomenon because whereas the expression of HLA-DR molecules is decreased, the capacity for presenting glycolipids through CD1d expression is higher in BI patients. None of these effects could differentiate between BI patients with or without pneumonia.

### Specific activation of iNKT cells by PBMCs from BI patients

**Fig. 2:**
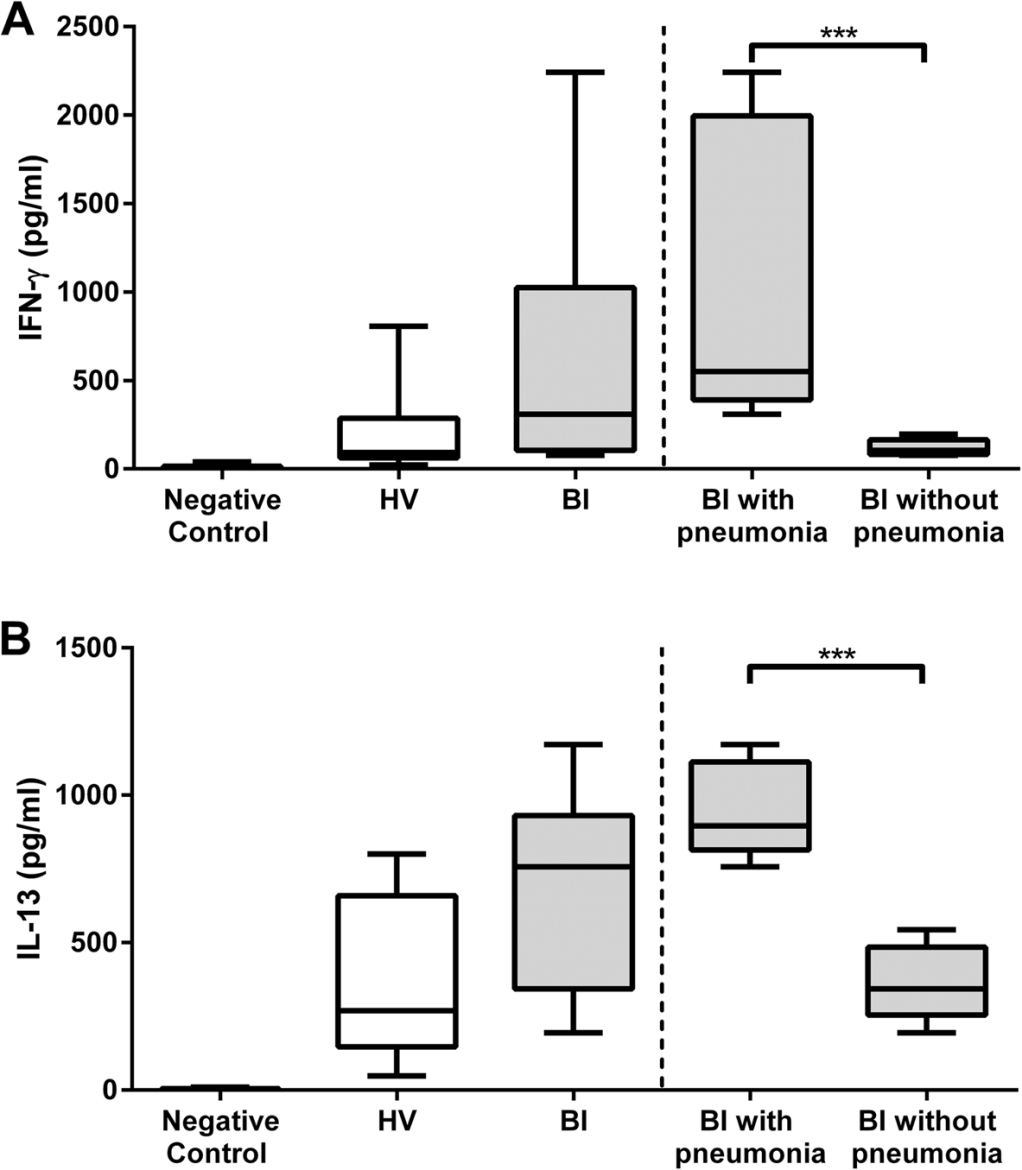
Cytokine secretion of iNKT cells activated by α-GalCer loaded on PBMCs from BI patients. iNKT cells isolated from a healthy donor were stimulated by PBMCs loaded with α-GalCer from 10 healthy volunteers (HV) (white boxes) or from BI patients (gray boxes), 10 with pneumonia and 9 without pneumonia. Cytokines secretions were analyzed by ELISA after 48 h. **a** IFN-γ. **b** IL-13. Data are shown as the concentration of cytokines in pg/ml and are representative of two independent experiments. Negative controls indicate cytokine secretion in the absence of α-GalCer. Right panels show the data obtained for BI patients when split into those who declared pneumonia and those who did not. Top and bottom whiskers represent the extreme values; boxed area represents the 25th percentile, the median, and the 75th percentile (***p < 0.001; statistical analysis, Mann-Whitney test)

The elevated level of CD1d suggested an increased ability of PBMCs from BI patients to activate iNKTs. Specific activation of iNKT cells occurs following the presentation of glycolipid antigens by APCs loaded onto the CD1d molecule. We thus investigated IFN-γ and IL-13 secretions following activation of iNKT cells isolated from HV using PBMCs loaded with α-GalCer as APCs from either HV or BI patients. There was a trend toward higher production of IFNγ and IL-13 in the BI group as compared with the HV group (Fig. 2a and b, left panels). Yet, the large dispersal of the values obtained from the patient group prompted us to compare the patients with or without pneumonia. Strikingly, we observed a much stronger concentration of both IFN-γ and IL-13 in BI patients with pneumonia (Fig. 2a and b, right panels). This indicates that the early iNKT antigen-specific response could predict patients with subsequent infection. It also indicates that contrary to the impaired expression of HLA-DR, the antigen presentation system through CD1d remains functional.

### Alterations of iNKT cells in BI patients

**Fig. 3:**
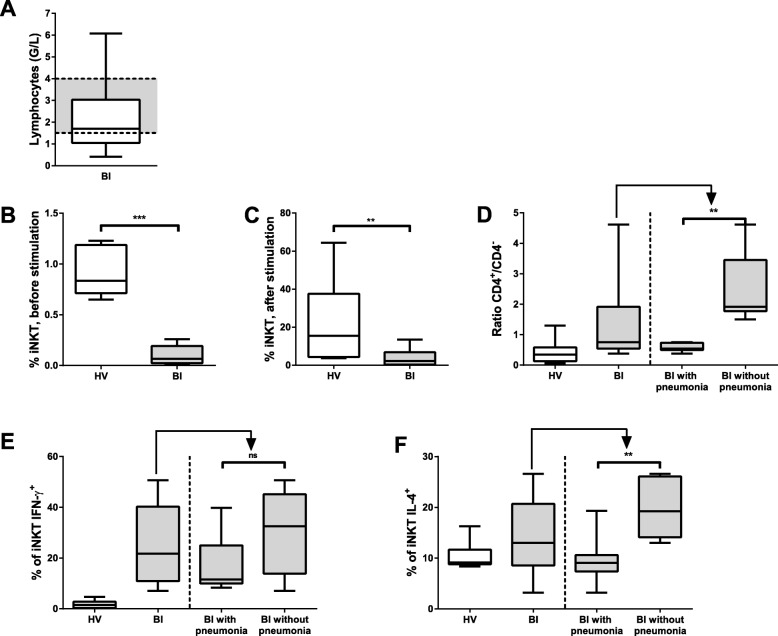
Analysis of lymphocytes population in PBMC by flow cytometry. Box and whisker plot summarizing the number of lymphocytes in BI patients. The gray area shows the normal range in healthy population (1.5–4 × 10^9^ cells/L) (**a**). After α-GalCer expansion, iNKT cell populations were compared. PBMCs from 10 healthy volunteers (HV) (white boxes) or PBMC from BI (gray boxes), 9 with pneumonia, and 8 without pneumonia were cultured in medium containing α-GalCer and IL-2 for 10 days to induce iNKT cell expansion. Boxes and whisker plots summarizing the percentage of iNKTs in the CD3^+^ compartment before stimulation (**b**) or after stimulation (**c**), the ratio of CD4^+^/CD4^−^ iNKT cells (**d**), the percentage of iNKT IFN-γ^+^ (**e**), and the percentage of iNKT IL-4^+^ (**f)**. Top and bottom whiskers represent the extreme values; boxed area represents the 25th percentile, the median, and the 75th percentile (**p < 0.01, ***p < 0.001; statistical analysis, Mann-Whitney test)

After having observed alterations in patients’ iNKT antigen-presenting cells, we investigated the iNKT cells themselves. We first quantified circulating lymphocytes and observed a trend toward lymphopenia at 1 day post-BI (Fig. 3a and Additional file 7: Figure S5) along with a dramatic decrease of the % of iNKT cells (Figs. 3b and Additional file 7: Figure S5). Considering the small number of cells, we amplified iNKT cells to analyze their phenotype. To this aim, PBMCs were cultured for 10 days in the presence of α-GalCer. In BI, the iNKT proportion remained significantly lower than in HV after amplification (Fig. 3c). We first focused on subsets of iNKT cells. We observed a higher CD4+/CD4− ratio of the iNKT cells from BI patients and an even higher ratio in patients who did not develop pneumonia (Fig. 3d). CD4+ iNKT cells are known to have a T_h_2 phenotype characterized by a strong IL-4 secretion [22]. The proportion of iNKT cells secreting IFN-γ was much higher in patients than in HV following expansion and activation (Fig. 3e). These effects occurred independently of the patients’ status regarding the occurrence of pneumonia. But interestingly, consistent with their high CD4+/CD4- ratio, iNKT cells from patients who did not develop pneumonia presented a higher frequency of IL-4 positive cells (Fig. 3f).

### Patients’ serum factors decrease the specific activity of iNKT cells

**Fig. 4:**
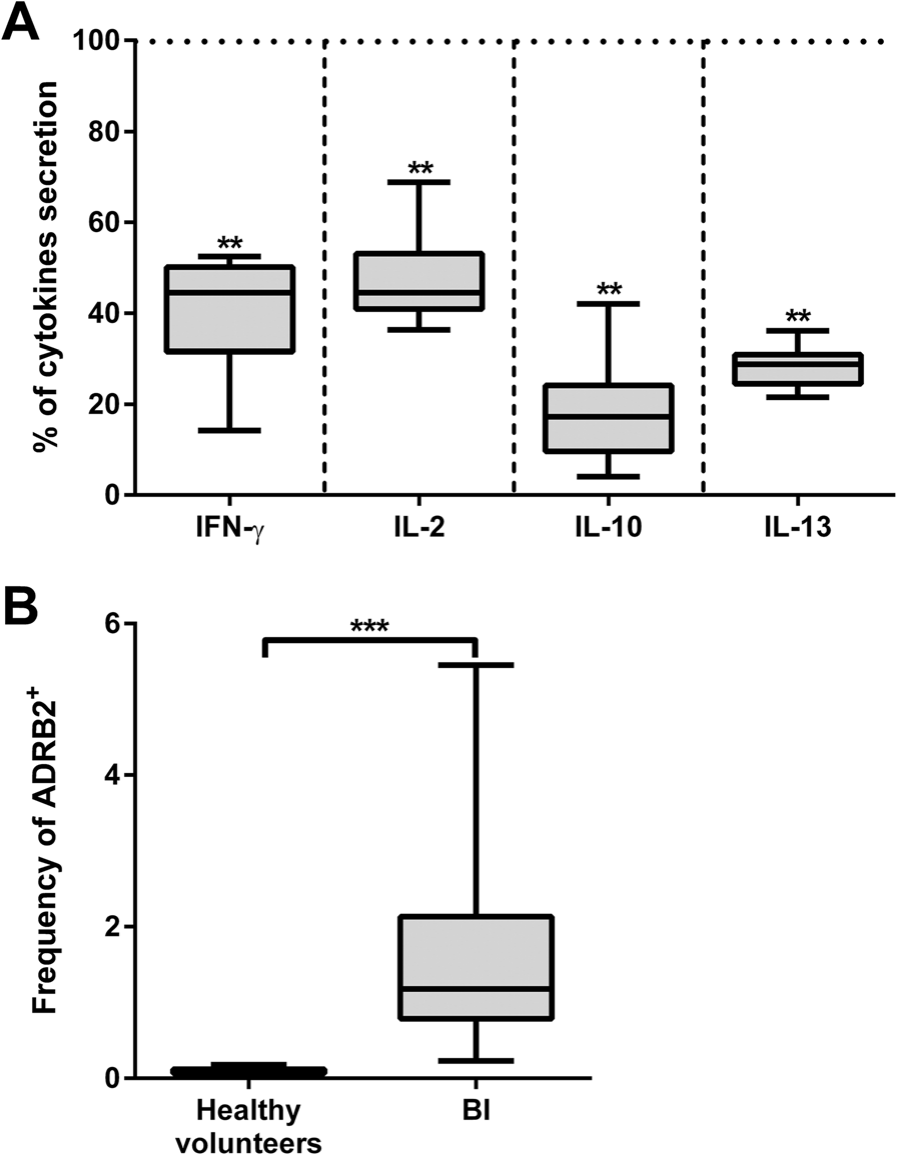
Cytokine secretion after specific activation of iNKT cells in BI patients’ sera and adrenergic receptor β2 (ADRB2) expression. **a** iNKT cells isolated from a healthy donor were stimulated with α-GalCer loaded on PBMCs from 8 healthy volunteers (HV), in medium containing pooled serum from 40 HV or sera from 20 BI patients. Cytokines were measured in supernatants by ELISA after 48 h. Data are shown as the percentage of each secreted cytokine when BI’s serum was used, 100% corresponding to the secretion in the presence of HV serum (horizontal dashed bar). Graphic is representative of two independent experiments. **b** Expression of the ADRB2 on the surface of CD3^+^ lymphocytes. Data are shown as the percentage of positive cells for 5 HV and 10 BI patients. Top and down whiskers represent the 25th percentile, the median, and the 75th percentile (**p < 0.01, ***p < 0.001; statistical analysis, Mann-Whitney test)

We then aimed at determining the presence of factors in sera from BI patients that may alter the specific activity of iNKTs cells. We co-cultured purified iNKTs from HV with PBMCs from 8 HV as APCs, loaded with α-GalCer either in the presence of culture medium containing pooled serum from 40 HV or pooled serum from 10 BI patients. Considering that HV condition represents the maximum of cytokine secretion (100%), iNKT cell activation in the presence of BI patients’ serum led to a significantly weaker secretion of all tested cytokines (IFN-γ, IL-2, IL-10, and IL-13) (Fig. 4a). These results indicate the presence of mediator(s) in the serum of patients that decrease the specific activation of iNKT cells with no difference between patients with or without pneumonia. While exploring the potential involvement of catecholamines in the phenomenon, we observed a clear increase of adrenergic receptor B2 at the surface of T lymphocytes from BI patients compared with its very low expression on those from HV (Fig. 4b and Additional file 8: Figure S6).

## Discussion

Cerebral ischemia after severe TBI or SAH is frequent and is a major factor of central nervous system injury-induced immune deficiency syndrome [5–7].

The specificity of BI as compared to other acute pathologies like septic shock relies on the central nervous system modulation of the immune system. This input is processed via 3 main pathways HPA axis and sympathetic and parasympathetic immune system. The sympathetic storm after BI is a well-known cause of systemic immunosuppression through the extensive sympathetic innervation of immune organs and the expression of adrenergic receptors on almost all leukocytes. The vagal “cholinergic pathway” displays anti-inflammatory effects through a direct effect of acetyl-choline on immune cells. The impairment of the adrenal axis is frequent and also induces a state of immunosuppression rendering the BI patient prone to secondary infections. These neuro-hormonal modifications may lead to a profound metabolic shift of immune cells through either a state of tolerance (risk of secondary infections) or trained immunity.

We found IL-10 in the serum of BI patients, but not in the serum from healthy volunteers. We additionally observed that the patients’ PBMCs presented a near complete loss of cytokine production after a mitogenic stimulation with IL-2. Interestingly, the effect was visible both for IFN-γ, a pro-inflammatory (T_h_1) cytokine, and IL-13, an anti-inflammatory (T_h_2) cytokine. These results are consistent with an early and deep alteration of the overall immune response. We next confirmed the strong downregulation of HLA-DR expression on patients’ monocytes [23] and as previously described [21], we additionally observed that this downregulation also existed in the B cell compartment. Overall, these data indicate that patients suffer from a systemic immunodepression.

Focusing on the iNKT compartment, we then unexpectedly observed a clear over-expression of CD1d both in monocytes and B cells. It was visible regardless of whether the patients would later develop pneumonia. This was the exact opposite of what was found for HLA-DR. CD1d is the non-classical MHC-I molecule involved in antigen presentation to iNKTs, suggesting a potential increased potency of patient’s APCs to activate iNKT cells in an antigen-dependent manner. Following specific activation of iNKT cells using PBMCs from patients or HV, a significant increase of cytokine secretion IFN-γ and IL-13 was observed, but only in BI patients that contracted pneumonia several days after BI. This indicates that the APCs of patients who are able to control infection possess other characteristics that modulate specific iNKT activity despite increased CD1d expression on their APCs. Interestingly, some suppressive cell subsets express high levels of CD1d such as monocytic myeloid-derived suppressor cells or regulatory B cells (Breg). In the latter case, it has been shown in mice that CD1d-lipid presentation by Breg cells induces secretion of IFN-γ by iNKT cells, thereby contributing to downregulation of Th1 and Th17 responses [24, 25]. The increased CD1d expression observed in BI patients may therefore reflect an increased frequency of CD1d-expressing regulatory cells and an iNKT-dependent endogenous glycolipid presentation that would lead to an increase in regulatory cytokines secretion through subsets, such as Breg cells, specifically increased in patients who will develop pneumonia. Further studies will be needed to more precisely define the phenotype of cells that over-express CD1d in BI patients.

We then sought to analyze the fate of patients’ iNKT cells and observed a major decrease of their frequency in the periphery. An earlier study highlighted a decrease up to 40% of the major lymphocytes subsets 1 day after stroke [26]. The near-complete disappearance of iNKT cells among PBMCs is a more important alteration in light of the relatively contained lymphopenia observed in our cohort. After expansion in the presence of the specific glycolipid α-GalCer, patients’ iNKT cells were able to produce IFN-γ that represents an iNKT activation marker. To determine if iNKT cells from patients can secrete as much IFN-γ following specific activation as those from HV, a detailed kinetic analysis would be required since the lower IFN-γ secretion that we observed for the healthy donors in our experimental conditions might be due to exhaustion after a 10-day stimulation. We observed an increased frequency of CD4^+^ iNKT cells in patients who did not develop infection. This cell population is considered as a T_h_2 sub-population [12], in agreement with the high level of IL-4^+^ iNKT cells also observed for this sub-group of patients. This increase in IL-4+ iNKT cells among patients who did not develop pneumonia is rather surprising. One possible explanation is that CD4+ iNKT cells appear to represent a less mature subset of iNKT cells [13]. It is thus possible that exhaustion of the most mature iNKT cell pool was more pronounced in patients who developed pneumonia than in those who did not. Beyond the type of response (pro- or anti-inflammatory), the current results clearly indicate that an altered reactivity of iNKT cells to their usual ligands is probably tightly related to the occurrence of secondary infections.

BI is largely reported to induce activation of the sympathetic nervous system leading to the release of numerous molecules in circulation that impair immune function [6]. Several studies focused on the central implication of catecholamines to promote systemic immunodepression [6, 9]. Although having highlighted serum factors able to downregulate cytokine secretion, we failed to revert the phenomenon after treatment with antagonist of catecholamines, propranolol (data not shown). The serum factors responsible for the downregulation of the immune response thus remain to be characterized. Nonetheless, involvement of the adrenergic pathway cannot be excluded. Indeed, analysis of the cell surface expression of the beta-2 adrenergic receptor on T lymphocytes revealed a marked overexpression in BI patients. Our results suggest that this could represent a potent stress marker of the lymphocytes of BI patients and potentially of patients with related immunodepression**.**

Some limitations deserve comments. First, the patterns of immune dysfunction described here could be general patterns observed in other populations of ICU patients. We aimed to perform a pathophysiological study with some complex experiments; this explains the relatively small number of patients studied and the lack of power analysis or logistic regression analysis for the comparison between infected and non-infected patients.

## Conclusions

Overall, the current results indicate that iNKT cells could participate to the post BI immune response alongside other factors. CD1d expression and beta-2 adrenergic receptor may be new candidate markers of immunodepression, showing increased rather than decreased expression. The decreased capacity to present antigens is not a generalized phenomenon, and we demonstrate for the first time that the capacity for presenting glycolipids to iNKT cells through CD1d expression on monocytes is higher in BI patients.

## Additional files

Additional file 1: **Table S1.** Comparative characteristics of TBI versus SAH patients. (DOCX 15 kb)

Additional file 2: **Table S2.** Pathogens involved in nosocomial pneumonia. (DOCX 14 kb)

Additional file 3: **Figure S1.** Presence of IL-10 in serum from individual BI patients. IL-10 was measured in serum from 10 healthy volunteers (HV) and 20 BI patients by ELISA. Results are shown as the concentration of cytokine in pg/ml. IFN-γ, TGF-β, IL-2, IL-4, IL-10, IL-12, and IL-13 could not be detected in the serum of either patients or healthy volunteers. (TIF 41 kb)

Additional file 4: **Figure S2.** Alteration of PBMCs response in BI patients. PBMCs from 10 HV or from 19 BI patients were cultured in complete medium containing 10% pooled sera from 40 healthy donors. Concentrations of IFN-γ (left panel) and IL-13 (right panel) in culture supernatants were measured by ELISA after a 48-h stimulation with 200 μg/ml recombinant IL-2. Data are shown as the concentration in pg/ml of cytokines and are representative of 2 independent experiments. Negative controls represent results obtained in absence of IL-2 stimulation. Top and bottom whiskers represent the extreme values; boxed area represents the 25th percentile, the median, and the 75th percentile. (**p < 0.01, ***p < 0.001; statistical analysis, Mann-Whitney test). (TIF 97 kb)

Additional file 5: **Figure S3.** Monocyte and B cell gating strategy. Representative density plots illustrating the gating strategy used to analyze phenotypes of CD19^+^lymphocytes and CD14+ monocytes. (TIF 129 kb)

Additional file 6: **Figure S4.** Expression of HLA-DR on monocytes and B cells. The membrane marker, HLA-DR, was analyzed on stored CD14+ monocytes and CD19+ lymphocytes from 10 individual healthy volunteers (HV) and 20 individual BI patients. Data are shown as the ratio of median fluorescence intensity (RFI) obtained with the specific antibodies to those obtained with their associated control isotypes. Top and bottom whiskers represent the extreme values; boxed area represents the 25th percentile, the median, and the 75th percentile. (***p < 0.001; statistical analysis, Mann-Whitney test). (TIF 96 kb)

Additional file 7: **Figure S5.** iNKT cell gating strategy. Representative density plots illustrating the gating strategy used to analyze iNKT based on CD3 and iTCR expression and their cytokines secretions after intra-cellular staining. (TIF 128 kb)

Additional file 8: **Figure S6.** Lymphocyte gating strategy. Representative density plots illustrating the gating strategy used to analyze phenotypes of CD3+ lymphocytes from patients or HV. (TIF 89 kb)

## Data Availability

The datasets are available from the corresponding author on request with justification.

## Abbreviations

ADRB2: Adrenergic receptor β2
APC: Antigen-presenting cells
HV: Human volunteers
IFN-γ: Interferon gamma
IL-10: Interleukin 10
IL-12: Interleukin 12
IL-2: Interleukin 2
iNKT: Invariant natural killer cells
PBMC: Peripheral blood mononuclear cells
PHA: Phytohemagglutinin
RPMI: Roswell Park Memorial Institute, medium for cells culture
SAH: Spontaneous Sub-Arachnoid Hemorrhage
TBI: Traumatic brain injury
TCR: T cell receptor
Th1: Lymphocyte T helper type 1
Th2: Lymphocyte T helper type 2
α-GalCer: α-Galactosylceramide

## References

[1] Asehnoune K, Seguin P, Allary J (2014). Hydrocortisone and fludrocortisone for prevention of hospital-acquired pneumonia in patients with severe traumatic brain injury (Corti-TC): a double-blind, multicentre phase 3, randomised placebo-controlled trial. Lancet Respir Med.

[2] Roquilly A, Mahe PJ, Seguin P (2011). Hydrocortisone therapy for patients with multiple trauma: the randomized controlled HYPOLYTE study. JAMA.

[3] Roquilly A, Feuillet F, Seguin P (2016). Empiric antimicrobial therapy for ventilator-associated pneumonia after brain injury. Eur Respir J.

[4] Bronchard R, Albaladejo P, Brezac G (2004). Early onset pneumonia: risk factors and consequences in head trauma patients. Anesthesiology.

[5] Meisel C, Schwab JM, Prass K (2005). Central nervous system injury-induced immune deficiency syndrome. Nat Rev Neurosci.

[6] Prass K, Meisel C, Höflich C (2003). Stroke-induced immunodeficiency promotes spontaneous bacterial infections and is mediated by sympathetic activation reversal by poststroke T helper cell type 1-like immunostimulation. J Exp Med.

[7] Deknuydt F, Roquilly A, Cinotti R (2013). An in vitro model of mycobacterial granuloma to investigate the immune response in brain-injured patients. Crit Care Med.

[8] Hinson HE, Sheth KN (2012). Manifestations of the hyperadrenergic state after acute brain injury. Curr Opin Crit Care.

[9] Wong CHY, Jenne CN, Lee W-Y (2011). Functional innervation of hepatic iNKT cells is immunosuppressive following stroke. Science.

[10] Porcelli S, Yockey C, Brenner M (1993). Analysis of T cell antigen receptor (TCR) expression by human peripheral blood CD4-8- alpha/beta T cells demonstrates preferential use of several V beta genes and an invariant TCR alpha chain. J Exp Med.

[11] Bendelac A, Lantz O, Quimby M (1995). CD1 recognition by mouse NK1+ T lymphocytes. Science.

[12] Montoya CJ, Pollard D, Martinson J (2007). Characterization of human invariant natural killer T subsets in health and disease using a novel invariant natural killer T cell-clonotypic monoclonal antibody, 6B11. Immunology.

[13] Liu J, Hill BJ, Darko S, et al. The peripheral differentiation of human natural killer T cells. Immunol Cell Biol. 2019; in press. 10.1111/imcb.12248. 10.1111/imcb.12248 PMC6767057 30875134

[14] Brennan PJ, Brigl M, Brenner MB (2013). Invariant natural killer T cells: an innate activation scheme linked to diverse effector functions. Nat Rev Immunol.

[15] American Thoracic Society (2005). Infectious Diseases Society of America: guidelines for the management of adults with hospital-acquired, ventilator-associated, and healthcare-associated pneumonia. Am J Respir Crit Care Med.

[16] Asehnoune K, Seguin P, Lasocki S (2017). Extubation success prediction in a multicentric cohort of patients with severe brain injury. Anesthesiology.

[17] Connolly ES, Rabinstein AA, Carhuapoma JR (2012). Guidelines for the management of aneurysmal subarachnoid hemorrhage: a guideline for healthcare professionals from the American Heart Association/American Stroke Association. Stroke.

[18] Brain Trauma Foundation, American Association of Neurological Surgeons, Congress of Neurological Surgeons: guidelines for the management of severe traumatic brain injury. J Neurotrauma 2007; 24 Suppl 1:S1–106. 10.1089/neu.2007.9999 17511534

[19] Hunault J, Diswall M, J-CC F (2012). 3-fluoro- and 3,3-difluoro-3,4-dideoxy-KRN7000 analogues as new potent immunostimulator agents: total synthesis and biological evaluation in human invariant natural killer T cells and mice. J Med Chem.

[20] Roquilly A, David G, Cinotti R (2017). Role of IL-12 in overcoming the low responsiveness of NK cells to missing self after traumatic brain injury. Clin Immunol Orlando Fla.

[21] Chenouard A, Chesneau M, Braza F (2015). Phenotype and functions of B cells in patients with acute brain injuries. Mol Immunol.

[22] Gumperz JE, Miyake S, Yamamura T (2002). Functionally distinct subsets of CD1d-restricted natural killer T cells revealed by CD1d tetramer staining. J Exp Med.

[23] Lukaszewicz A-CC, Grienay M, Matthieu R-R (2009). Monocytic HLA-DR expression in intensive care patients: interest for prognosis and secondary infection prediction. Crit Care Med.

[24] An B, Lim J-Y, Jeong S (2018). CD1d is a novel cell-surface marker for human monocytic myeloid-derived suppressor cells with T cell suppression activity in peripheral blood after allogeneic hematopoietic stem cell transplantation. Biochem Biophys Res Commun.

[25] Oleinika K, Rosser EC, Matei DE (2018). CD1d-dependent immune suppression mediated by regulatory B cells through modulations of iNKT cells. Nat Commun.

[26] De Raedt S, De Vos A, Van Binst A-MM (2015). High natural killer cell number might identify stroke patients at risk of developing infections. Neurol Neuroimmunol Neuroinflamm.

