# Supplementary material for: Alterations of the iNKT cell compartment in brain-injured patients
Source: Crit Care. 2019 Jun 28;23:234. doi: 10.1186/s13054-019-2518-2 (PMC6599321; doi:10.1186/s13054-019-2518-2)
Supplement: Supplementary file 1 — Table S1. Comparative characteristics of TBI versus SAH patients. (DOCX 15 kb) [file 13054_2019_2518_MOESM1_ESM.docx]

**Table S1** Comparative characteristics of TBI versus SAH patients

| Demographic data and outcomes | Total  (n=33) | TBI  (n=20), 61% | ASH (n=13), 39% | P value |
| --- | --- | --- | --- | --- |
| Age (years) | 46 [26-55] | 42 [25-57] | 50 [42-55] | 0.4947 |
| Male, *n (%)* | 26 (79%) | 17 (85%) | 9 (69%) | 0.3926 |
| Initial GCS | 4 [4-8] | 6 [4-8] | 5 [3-8] | 0.7354 |
| Barbiturate, *n (%)* | 7 (21%) | 7 (35%) | 0 (0%) | 0.0163 |
| Corticotherapy, *n (%)* | 1 (3%) | 1 (5%) | 0 (0%) | n/a |
| Acute respiratory distress syndrome, *n(%)* | 6 (18%) | 2 (10%) | 4 (31%) | 0.1824 |
| Decompressive Cranectomy, *n*  *(%)* | 4 (12%) | 3 (15%) | 1 (8%) | n/a |
| Duration of mechanical ventilation (days) | 11 [7-19] | 10.5 [8-17] | 17 [6-20] | 0.9637 |
| ICU lenght of stay (days) | 15 [10-26] | 14.5 [10.75-20] | 20 [9-31] | p=0.7366 |
| Death in ICU, *n, (%)* | 6 (18%) | 4 (20%) | 2 (15%) | n/a |
| Pneumonia, *n, (%)* | 18 (55%) | 9 (45%) | 9 (69%) | 0.2844 |

Data are given as the median [interquartile range] or *n* (%); P<0.05 is considered as statistically significant.

ICU: intensive care unit; GCS : Glasgow Coma Scale; TBI: Traumatic Brain Injury; ASH : Aneurysmal subarachnoid haemorrhage; n/a: not applicable
