# Supplementary material for: Alterations of the iNKT cell compartment in brain-injured patients
Source: Crit Care. 2019 Jun 28;23:234. doi: 10.1186/s13054-019-2518-2 (PMC6599321; doi:10.1186/s13054-019-2518-2)
Supplement: Supplementary file 2 — Table S2. Pathogens involved in nosocomial pneumonia. (DOCX 14 kb) [file 13054_2019_2518_MOESM2_ESM.docx]

**Table S2**

Pathogens involved in nosocomial pneumonia

| Pathogens involved, n (%) | Nosocomial pneumonia (n=18) |
| --- | --- |
| Methicillin-sensitive *Staphylococcus aureus* | 5 (28%) |
| *Streptococus pneumoniae* | 3 (17%) |
| *Escherichia coli* | 3 (17%) |
| *Haemophius influenzae* | 2 (11%) |
| *Pseudomonas aeroginosa* | 1 (6%) |
| *Proteus mirabilis* | 1 (6%) |
| Other Gram negative bacteria | 1 (6%) |
| Polymicrobial pneumonia | 3 (17%) |
| Unknown | 1 (6%) |

Among the 33 brain-injured patients, there were 20 episodes of nosocomial pneumonia involving a total of 18 patients.
